# Association between prior hospitalization and nasal carriage of methicillin-resistant *Staphylococcus aureus* (MRSA) in community-dwelling U.S. adults: Evidence from NHANES

**DOI:** 10.1097/MD.0000000000043987

**Published:** 2025-08-15

**Authors:** Fengling Zhou, Yumei Dong, Lei Yu, Shaomin Chen, Li Tan

**Affiliations:** a Department of Disease Control and Prevention, General Hospital of Central Theater Command, Wuhan, Hubei, China.

**Keywords:** carriage, community-dwelling, hospitalization, MRSA, NHANES

## Abstract

While institution-based studies have established an association between prior hospitalization and methicillin-resistant *Staphylococcus aureus* (MRSA) nasal carriage, this relationship remains unevaluated in community-dwelling adults explicitly excluded from institutionalized populations. The objective of this study was to investigate the potential relationship between prior hospitalization and MRSA nasal carriage in adults residing in non-institutionalized community settings. This cross-sectional analysis included 9512 community-dwelling U.S. adults from the National Health and Nutrition Examination Survey, 2001 to 2004, defining prior hospitalization as self-reported overnight admission within the past 12 months to classify participants into hospitalized and non-hospitalized groups. Logistic regression and inverse probability of treatment weighting using the propensity score analyses were utilized to examine the association between prior hospitalization and MRSA nasal carriage. The mean age of participants was 49.65 ± 19.23 years, with 4966 being female. MRSA nasal carriage was identified in 3.13% (38 out of 1214) of participants in the hospitalized group, compared to 1.10% (91 out of 8298) in the non-hospitalized group. After adjusting for multiple covariates, prior hospitalization was associated with a significantly higher odds of MRSA nasal carriage (odds ratio: 2.03, 95% confidence intervals: 1.35–3.05, *P* < .001). Propensity score analyses corroborated these findings, yielding an odds ratio of 2.06 (95% confidence intervals: 1.37–3.10, *P* < .001). These results were consistent across multiple sensitivity analyses. Our study found that prior hospitalization within the past 12 months was associated with an increased risk of MRSA nasal carriage in community-dwelling U.S. adults. However, a limitation of this study is the unavailability of nasal culture data before hospital admission, which precludes determining whether MRSA carriage preceded hospitalization. Further studies are needed to confirm this association.

## 1. Introduction

Nasal carriage of methicillin-resistant *Staphylococcus aureus* (MRSA) remains a critical public health concern due to its potential to progress from asymptomatic states to severe infections.^[1]^ While previous studies have identified multiple clinical and environmental factors associated with MRSA carriage in institutionalized populations (e.g., inpatient populations or occupation-specific groups),^[2–4]^ the factor–carriage associations in non-institutionalized, community-dwelling adults remain comparatively understudied. A representative case is prior hospitalization: while this exposure exhibits measurable associations with MRSA carriage in institutionalized populations,^[5,6]^ such relationships in exclusively community-based settings have not been comprehensively analyzed.

Prior hospitalization is strongly linked to MRSA carriage in inpatient studies. A prospective multicenter study of patients admitted to German rehabilitation centers found that prior hospitalization within the preceding 6 months was a risk factor for MRSA carriage at admission.^[7]^ Similarly, U.S. research focused on hospitalized adults found that those with a hospitalization history exceeding 1 year prior to admission had elevated MRSA carriage compared to individuals without such exposure.^[8]^ Beyond inpatient studies, this association is also observable in occupation-specific populations as exemplified by elevated colonization rates among military personnel with recent hospitalization histories.^[9]^ However, current evidence originates almost entirely from institutionalized populations. Whether hospitalization history is similarly associated with MRSA carriage in non-institutionalized, community-dwelling adults remains to be investigated.

The National Health and Nutrition Examination Survey (NHANES) excludes individuals residing in any institutional group quarters (e.g., hospitals, nursing facilities, correctional institutions or military barracks), documenting community-dwelling residents in the civilian non-institutionalized population.^[10]^ This exclusion allows analysis of the association between prior hospitalization and MRSA carriage to remain free from confounding by institutional residency. Despite these design features explicitly excluding institutionalized populations, the NHANES resource remains underutilized in systematically assessing this association among community-dwelling adults exclusively.

We hypothesized that prior hospitalization within the past 12 months would be associated with nasal carriage of MRSA among adults in community settings. Leveraging the NHANES (2001–2004) exclusion of institutionalized populations via enrollment stratification, we conducted a cross-sectional analysis of non-institutionalized U.S. adults. Hereby, we aimed to evaluate this association and explore its manifestation specifically in community-dwelling adults distinct from institutionalized populations.

## 2. Methods

### 2.1. Study participants and design

**Figure 1.**
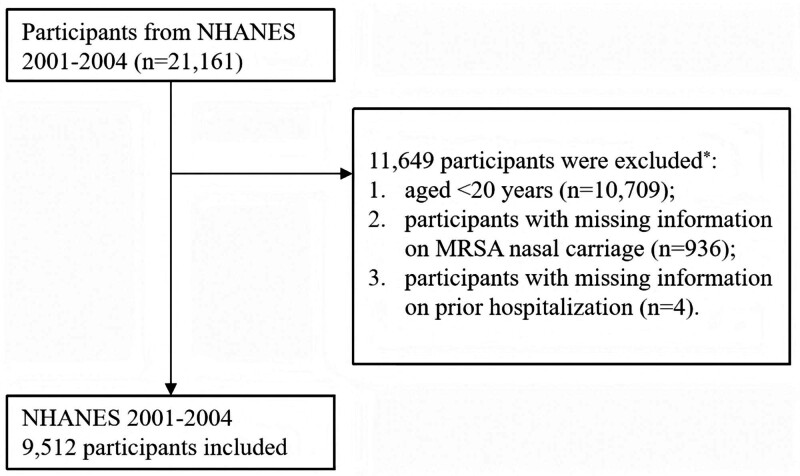
Flowchart of participant exclusion process. * Subjects selected after each step. MRSA = methicillin-resistant *Staphylococcus aureus*, NHANES = National Health and Nutrition Examination Survey.

Data on community-dwelling adults were obtained from NHANES, established by the National Center for Health Statistics under the Centers for Disease Control. This investigation incorporated a stratified multistage probabilistic design to achieve nationwide coverage of non-institutionalized U.S. residents. The current study used the datasets from 2 NHANES survey cycles, 2001 to 2002 and 2003 to 2004, during which *S aureus* nasal swab cultures were examined and recorded. The datasets are available on the NHANES website at https://wwwn.cdc.gov/nchs/nhanes/default.aspx. The National Center for Health Statistics Ethics Review Board of the National Center for Health Statistics provided ethical approval for all NHANES protocols. Participants provided written informed consent before data collection commenced. This secondary analysis utilized publicly available, fully de-identified data, therefore additional institutional ethics approval was not necessary. We screened 9512 eligible participants according to the following exclusion criteria: aged < 20 years; missing information on MRSA nasal carriage; missing information on self-reported prior hospitalization. The detailed screening process is shown full in Figure 1.

### 2.2. Assessment of prior hospitalization

Prior hospitalization was assessed by asking participants whether they had stayed overnight as admitted patients in a hospital during the past 12 months (excluding emergency department stays).^[11,12]^ Respondents answering “Yes” were classified as hospitalized; those answering “No” were classified as non-hospitalized.

### 2.3. Assessment of MRSA nasal carriage

Nasal swab specimens collected from NHANES 2001 to 2004 participants were processed according to standardized protocols.^[13,14]^ After initial culture on mannitol salt agar under protocol-specified incubation, colonies suggestive of *S aureus*, characterized by mannitol fermentation and yellow/gold morphology, were subcultured onto blood agar plates. Confirmation involved Staphaurex latex agglutination testing and supplemental tube coagulase assays. Oxacillin disk diffusion was then applied to confirmed *S aureus* isolates, with methicillin resistance defined as an inhibition zone diameter ≤ 10 mm according to the Clinical and Laboratory Standards Institute criteria. All specimens were dichotomously classified as MRSA nasal carriage if resistant *S aureus* was detected or as non-carriage if no resistant isolates were identified.

### 2.4. Other covariates

Through well-designed interviews and examinations, NHANES collected information on sociodemographic characteristics, health status, and laboratory metrics. Covariates were selected based on previous literature and clinical expertise.^[14–18]^ The variables included in current study were age, sex (female or male), race (non-Hispanic White, non-Hispanic Black, and other race), marital status (never married, married/cohabiting, and widowed/divorced/separated), education (less than high school, high school or equivalent, and college or above).

The poverty income ratio (PIR), an index calculated by dividing family income by a poverty threshold based on family size, was reported by 3 categories^[19]^: ≤1.3, 1.3 to 3.5, and > 3.5. Smoking status was categorized as: never (<100 cigarettes), current (≥100 and currently smoking everyday), and former smoker (≥100, not current). Drinking was categorized dichotomously (yes/no) based on exceeding 12 annual drinks.^[20]^ Physical activity was stratified into 3 categories^[21]^: lightly active (<600 metabolic equivalents [MET]/week), moderately active (600–2400 MET/week), and highly active (>2400 MET/week). Body mass index (kg/m²) was categorized into 3 groups: <25.0, 25.0 to 29.9, and ≥ 30.0.

Diabetes was defined as meeting any of the following criteria^[20,22]^: self-reported diagnosis, current use of insulin or oral hypoglycemic agents, hemoglobin A1c ≥ 6.5%, fasting blood glucose ≥ 7.0 mmol/L, or 2-hour postprandial glucose ≥ 11.1 mmol/L. Hypertension was defined by meeting at least 1 criterion: self-reported diagnosis; current antihypertensive medication use; or systolic blood pressure ≥ 130 mm Hg and/or diastolic blood pressure ≥ 80 mm Hg.^[23]^ Cardiovascular disease (CVD) was determined through participant-reported history of heart failure, coronary heart disease, angina pectoris, or myocardial infarction. Asthma was identified based on participants’ affirmative response to the survey item: “Has a doctor or other health professional ever told you that you have asthma?” Current health status was determined through participant-reported assessments and categorized into 2 groups: excellent/very good/good or fair/poor.

Antibiotic use was defined as a binary variable (yes/no) based on self-reported prescription medication intake within the past 30 days collected through structured interviews.^[24]^ Reported medications, including both single-ingredient and multi-ingredient formulations containing ≥1 antibiotic agent, were processed through the Multum Lexicon database (Cerner Multum, Inc., Kansas City ), with variable and value names standardized according to the prescription medication-drug information (RXQ_DRUG) file specifications.^[25]^ Vitamin D status was determined using radioimmunoassay measurements subsequently standardized to liquid chromatography-tandem mass spectrometry-equivalent values, with serum 25-hydroxyvitamin D concentrations serving as the quantitative measurement. Participants were dichotomized into deficiency (<20 ng/mL) or adequacy (≥20 ng/mL) groups using the 25-hydroxyvitamin D cutoffs established in a prior investigation.^[26]^ Participants reported health insurance coverage status through self-identification, with affirmative responses categorizing them as insured and negative replies designating them as uninsured. Healthcare visits were categorized as 0, 1 to 2, or ≥ 3 encounters based on self-reported non-hospitalization medical consultations (clinic/emergency visits) during the preceding year.

### 2.5. Statistical analysis

Continuous variables were expressed as mean ± standard deviation, while categorical variables were summarized as frequency counts with corresponding percentages. Comparative analyses between hospitalized groups and non-hospitalized groups were performed using *t* test for normally distributed continuous data and chi-square test for categorical variables.

Logistic regression model analyses were employed. Effect estimates were expressed as odds ratio (OR) along with their respective 95% confidence intervals (CI). The selection of confounding variables was based on their association with the outcomes of interest or changes in effect estimates of more than 10%.^[27]^ Following a consideration of the clinical significance, adjustments were made for the following covariates: age (years), sex, race, marital status, PIR, smoking status, physical activity, diabetes, hypertension, CVD, asthma, current health status, and vitamin D status.

To mitigate the potential confounding effects, a propensity score (PS) approach was employed.^[28–30]^ Optimal full matching was conducted utilizing the R package MatchIt (maintained by Daniel Ho, Stanford University, Stanford ), with the distance metric specified as the PS derived from the generalized additive model. Subsequently, to estimate the treatment effect post-full matching, the generalized additive model with a logit link function was utilized. The estimation of standard errors and confidence intervals was carried out using cluster-robust standard errors, with pair membership as the cluster, facilitated by the vcovCL() function from the R package sandwich.

Sensitivity analysis was conducted to evaluate the robustness of our findings. Inverse probability of treatment weighting (IPTW) using the PS analyses were employed to explore the relationship between prior hospitalization and MRSA nasal carriage. Within the IPTW method, the predicted probabilities derived from the PS model were used to compute the IPTW.^[31]^ Dummy variables were implemented to indicate missing covariate values. The E-value was calculated to assess the potential impact of unmeasured confounders on the relationship between prior hospitalization and the risk of MRSA nasal carriage.^[32]^ The E-value quantifies the degree of unmeasured confounding necessary to nullify the observed association between prior hospitalization and MRSA nasal carriage. To further evaluate the robustness of our findings, we conducted stratified analyses using stratified Cox proportional hazards regression models. Interaction effects were assessed using likelihood ratio tests, which compared models with and without interaction terms.

Statistical analyses were performed utilizing R software (version 4.2.0) and EmpowerStats software (X&Y Solutions, Inc., Boston). The threshold for statistical significance was established at a *P* value of less than .05.

## 3. Results

### 3.1. Baseline characteristics of the participants

A total of 9512 adults aged ≥ 20 years from NHANES 2001 to 2004 were included (Table 1). The mean age was 49.65 ± 19.23 years, and 52.21% were female. Among participants, 1214 (12.76%) reported prior hospitalization, while MRSA nasal carriage was identified in 129 (1.36%) cases overall. Significant differences in sociodemographic characteristics, lifestyle behaviors, and health conditions were observed between hospitalized and non-hospitalized groups.

**Table 1:** Clinical and demographic baseline characteristics of the National Health and Nutrition Examination Survey participants according to prior hospitalization, 2001 to 2004.

Characteristics	Overall (n = 9512)	Prior hospitalization		
		Non-hospitalized (n = 8298)	Hospitalized (n = 1214)	*P* value
Age (yr)	49.65 ± 19.23	48.91 ± 18.92	54.73 ± 20.54	<.001
Sex, n (%)				<.001
Female	4966 (52.21)	4226 (50.93)	740 (60.96)	
Male	4546 (47.79)	4072 (49.07)	474 (39.04)	
Race, n (%)				.175
Non-Hispanic White	5014 (52.71)	4362 (52.57)	652 (53.71)	
Non-Hispanic Black	1834 (19.28)	1586 (19.11)	248 (20.43)	
Other race	2664 (28.01)	2350 (28.32)	314 (25.86)	
Marital status, n (%)				<.001
Never married	1529 (16.08)	1386 (16.71)	143 (11.79)	
Married/cohabiting	5878 (61.83)	5165 (62.28)	713 (58.78)	
Widowed/divorced/separated	2099 (22.08)	1742 (21.01)	357 (29.43)	
Education, n (%)				<.001
Less than high school	1350 (14.21)	1152 (13.90)	198 (16.34)	
High school or equivalent	3775 (39.75)	3246 (39.17)	529 (43.65)	
College or above	4373 (46.04)	3888 (46.92)	485 (40.02)	
PIR, n (%)				<.001
≤1.3	2501 (28.03)	2100 (26.95)	401 (35.55)	
1.3–3.5	3502 (39.26)	3045 (39.07)	457 (40.51)	
>3.5	2918 (32.71)	2648 (33.98)	270 (23.94)	
Smoking status, n (%)				<.001
Never smoker	4849 (50.99)	4257 (51.31)	592 (48.76)	
Current smoker	2130 (22.40)	1896 (22.85)	234 (19.28)	
Former smoker	2531 (26.61)	2143 (25.83)	388 (31.96)	
Drinking status, n (%)				<.001
No	5492 (62.27)	4649 (60.31)	843 (75.88)	
Yes	3327 (37.73)	3059 (39.69)	268 (24.12)	
Physical activity, n (%)				<.001
Lightly active	4615 (48.52)	3890 (46.88)	725 (59.72)	
Moderately active	3046 (32.03)	2727 (32.87)	319 (26.28)	
Very active	1850 (19.45)	1680 (20.25)	170 (14.00)	
BMI, n (%)				<.001
<25.0 kg/m^2^	2910 (31.78)	2558 (31.90)	352 (30.99)	
25.0–29.9 kg/m^2^	3324 (36.30)	2959 (36.90)	365 (32.13)	
≥30.0 kg/m^2^	2922 (31.91)	2503 (31.21)	419 (36.88)	
Diabetes, n (%)				<.001
No	8351 (87.79)	7397 (89.14)	954 (78.58)	
Yes	1161 (12.21)	901 (10.86)	260 (21.42)	
Hypertension, n (%)				<.001
No	4465 (46.94)	3974 (47.89)	491 (40.44)	
Yes	5047 (53.06)	4324 (52.11)	723 (59.56)	
CVD, n (%)				<.001
No	8584 (90.27)	7671 (92.47)	913 (75.27)	
Yes	925 (9.73)	625 (7.53)	300 (24.73)	
Asthma, n (%)				.001
No	8449 (88.93)	7403 (89.33)	1046 (86.16)	
Yes	1052 (11.07)	884 (10.67)	168 (13.84)	
Current health status, n (%)				<.001
Excellent/very good/good	6848 (77.55)	6129 (79.43)	719 (64.48)	
Fair/poor	1983 (22.45)	1587 (20.57)	396 (35.52)	
Antibiotic use, n (%)				<.001
No	8969 (95.37)	7872 (95.84)	1097 (92.18)	
Yes	435 (4.63)	342 (4.16)	93 (7.82)	
Vitamin D, n (%)				<.001
≥20 ng/mL	5640 (62.49)	4985 (63.19)	655 (57.61)	
<20 ng/mL	3386 (37.51)	2904 (36.81)	482 (42.39)	
Health insurance, n (%)				<.001
Insured	7625 (81.09)	6566 (80.07)	1059 (88.03)	
Uninsured	1778 (18.91)	1634 (19.93)	144 (11.97)	
Healthcare visits, n (%)				<.001
0	1449 (15.24)	1432 (17.26)	17 (1.40)	
1–2	4085 (42.96)	3844 (46.34)	241 (19.87)	
≥3	3974 (41.80)	3019 (36.40)	955 (78.73)	
MRSA, n (%)				<.001
No	9383 (98.64)	8207 (98.90)	1176 (96.87)	
Yes	129 (1.36)	91 (1.10)	38 (3.13)	

Data are expressed as mean ± standard deviation or frequencies (%).

The *P* value was calculated using Student *t* test for continuous variables and the Chi-square test for categorical variables.

Within the study of 9512 participants, the proportion of missing data for analyzed variables comprised 6 (0.06 %) for marital status, 14 (0.15 %) for education, 591 (6.21 %) for PIR, 2 (0.02 %) for smoking status, 693 (7.29 %) for drinking, 1 (0.01 %) for physical activity, 356 (3.74 %) for BMI, 3 (0.03 %) for CVD, 11 (0.12 %) for asthma, 681 (7.16 %) for current health status, 108` (1.14 %) for antibiotic use, 486 (5.11 %) for vitamin D, 109 (1.15 %) for health insurance, and 4 (0.04 %) for healthcare visits.

PIR = poverty income ratio, BMI = body mass index, CVD = cardiovascular disease, MRSA = methicillin-resistant *Staphylococcus aureus*.

### 3.2. Factors influencing the risk of MRSA nasal carriage analyzed by logistics regression

In univariate analyses (Table 2), prior hospitalization significantly associated with higher odds of MRSA nasal carriage (OR: 2.91, 95% CI: 1.99–4.28, *P* < .001). Additionally, older age, former smoker status, diagnosis of diabetes, CVD, and increased healthcare visits were significantly related to higher odds of MRSA nasal carriage.

**Table 2:** Unadjusted associations of baseline variables with methicillin-resistant *Staphylococcus aureus* nasal carriage.

Exposure		OR (95% CI)	*P* value
Prior hospitalization	Non-hospitalized	1	
	Hospitalized	2.91 (1.99, 4.28)	<.001
Age, years		1.03 (1.02, 1.04)	<.001
Sex	Female	1	
	Male	0.67 (0.47, 0.95)	.026
Race	Non-Hispanic White	1	
	Non-Hispanic Black	0.83 (0.53, 1.30)	.422
	Other race	0.50 (0.31, 0.80)	.004
Marital status	Never married	1	
	Married/cohabiting	0.73 (0.45, 1.18)	.202
	Widowed/divorced/separated	1.30 (0.78, 2.18)	.312
Education	Less than high school	1	
	High school or equivalent	0.89 (0.54, 1.47)	.655
	College or above	0.73 (0.44, 1.20)	.212
PIR	≤1.3	1	
	1.3–3.5	0.79 (0.53, 1.19)	.266
	>3.5	0.52 (0.32, 0.84)	.008
Smoking status	Never smoker	1	
	Current smoker	1.03 (0.63, 1.68)	.916
	Former smoker	2.09 (1.42, 3.07)	<.001
Drinking	No	1	
	Yes	0.65 (0.44, 0.98)	.039
Physical activity	Lightly active	1	
	Moderately active	0.54 (0.36, 0.83)	.004
	Very active	0.48 (0.28, 0.82)	.007
BMI (kg/m^2^)	<25.0	1	
	25.0–29.9	0.72 (0.46, 1.14)	.165
	≥30.0	1.12 (0.73, 1.71)	.603
Diabetes	No	1	
	Yes	2.21 (1.46, 3.34)	<.001
Hypertension	No	1	
	Yes	1.05 (0.74, 1.49)	.783
CVD	No	1	
	Yes	2.38 (1.54, 3.68)	<.001
Asthma	No	1	
	Yes	1.86 (1.19, 2.90)	.007
Current health status	Excellent/very good/good	1	
	Fair/poor	1.88 (1.28, 2.76)	.001
Antibiotic use	No	1	
	Yes	1.08 (0.47, 2.46)	.861
Vitamin D, ng/ml	≥20	1	
	<20	1.55 (1.08, 2.21)	.017
Health insurance	Insured	1	
	Uninsured	0.70 (0.42, 1.15)	.161
Healthcare visits	0	1	
	1–2	1.21 (0.64, 2.32)	.556
	≥3	2.33 (1.27, 4.30)	.007

BMI = body mass index, CI = confidence interval, CVD = cardiovascular disease, OR = odds ratio, PIR = poverty income ratio.

### 3.3. The association between prior hospitalization and MRSA nasal carriage

MRSA nasal carriage was detected in 38 participants (3.13%) in the hospitalized group, whereas 91 participants (1.10%) in the non-hospitalized group tested positive for MRSA. Table 3 delineated the associations between prior hospitalization and MRSA nasal carriage utilizing various analytical methodologies: crude analysis, multivariable analysis, and PS analyses. In the crude analysis, prior hospitalization was associated with a significantly higher odds of MRSA nasal carriage, with an OR of 2.91 (95% CI: 1.99–4.28, *P *< .001). In the multivariable analysis, after adjusting for multiple covariates, the OR was 2.03 (95% CI: 1.35–3.05, *P *< .001). Upon adjusting for the PS, the OR was 2.06 (95% CI: 1.37–3.10, *P *< .001), and with PS (smooth), the OR remained at 2.01 (95% CI: 1.34–3.01, *P *< .001).

**Table 3:** Associations between prior hospitalization and methicillin-resistant *Staphylococcus aureus* nasal carriage, as analyzed in the crude analysis, multivariable analysis, and propensity score analyses.

Analysis	MRSA nasal carriage	*P* value
No. of events/no. of participants at risk (%)		<.001
Hospitalized	38/1214 (3.13%)	
Non-hospitalized	91/8298 (1.10%)	
Crude analysis – odds ratio (95% CI)	2.91 (1.99, 4.28)	<.001
Multivariable analysis – odds ratio (95% CI)		
1: Adjust for all covariates	2.03 (1.35, 3.05)	<.001
2: Adjust for PS	2.06 (1.37, 3.10)	<.001
3: Adjust PS (smooth)	2.01 (1.34, 3.01)	<.001
Estimate of exposure effect using IPTW – odds ratio (95% CI)		
ATT	1.87 (1.20, 2.91)	.006
ATC	2.03 (1.29, 3.20)	.002
ATE	2.00 (1.30, 3.09)	.002

*Note for models*: 1: Adjusted for indicator of any missing, age (years; smooth), sex, race, marital status, poverty income ratio, smoking status, physical activity, diabetes, hypertension, cardiovascular disease, asthma, current health status and vitamin D status. 2: Propensity score estimated by indicator of any missing, age (years; smooth), sex, race, marital status, poverty income ratio, smoking status, physical activity, diabetes, hypertension, cardiovascular disease, asthma, current health status, and vitamin D status.

ATC = average treatment effect for control, ATE = average treatment effect for all, ATT = average treatment effect for treated, CI = confidence interval, IPTW = inverse probability of treatment weighting, MRSA = methicillin-resistant *Staphylococcus aureus*, PS = propensity score.

### 3.4. Stratified analysis for the association between prior hospitalization and MRSA nasal carriage

**Figure 2.**
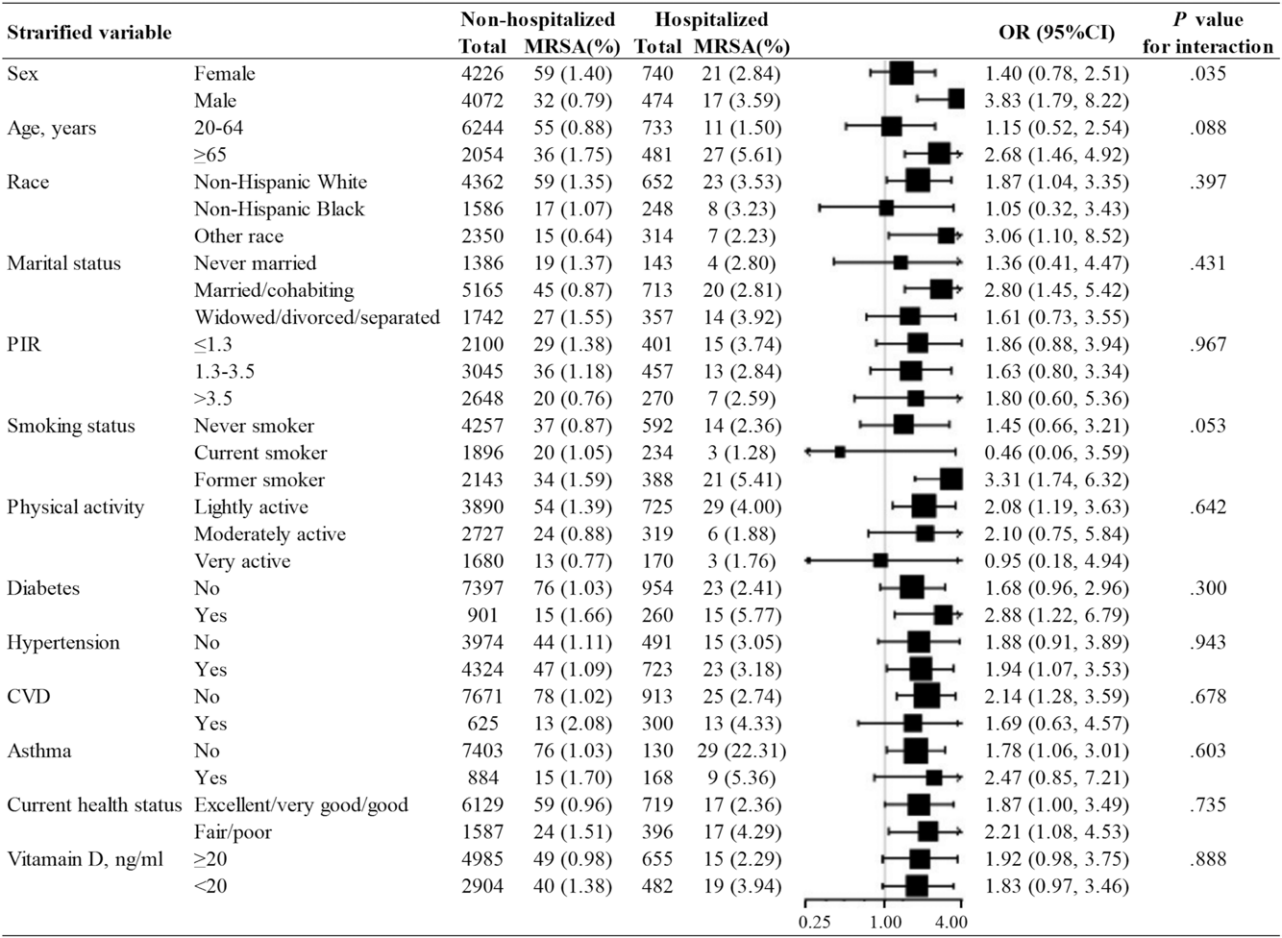
Stratified associations between prior hospitalization and MRSA nasal carriage. The multivariate model adjusted for age (years), sex, race, marital status, PIR, smoking status, physical activity, diabetes, hypertension, CVD, asthma, current health status and vitamin D status, except for the variable that is stratified. CI = confidence interval, CVD = cardiovascular disease, MRSA = methicillin-resistant *Staphylococcus aureus*, OR = odds ratio, PIR = poverty income ratio.

Considering the heterogeneity of the population, we conducted stratified regression analyses, as shown in the Figure 2. The results demonstrated that the association between prior hospitalization and MRSA nasal carriage was generally consistent across most subgroups, including age, race, PIR, and comorbidities such as diabetes and CVD. Notably, a significant interaction was observed with sex (*P* for interaction = .035). Among male, prior hospitalization was associated with higher odds of MRSA nasal carriage (OR: 3.83, 95% CI: 1.79–8.22, *P *< .001), while the association in female was no longer significant (OR: 1.40, 95% CI: 0.78–2.51, *P* = .266). No other subgroup analyses revealed statistically significant interactions (all *P* for interaction > .05).

### 3.5. Sensitive analysis

The sensitivity analysis produced results that were consistent with those of the primary analysis. As illustrated in Table 3, there were consistent associations between prior hospitalization and MRSA nasal carriage across various analytical approaches. Utilizing IPTW analyses, the average treatment effect for the treated was 1.87 (95% CI: 1.20–2.91, *P* = .006), the average treatment effect for the control was 2.03 (95% CI: 1.29–3.20, *P* = .002), and the average treatment effect for all was 2.00 (95% CI: 1.30–3.09, *P* = .002). The findings from crude, multivariable, and PS analyses consistently demonstrated that a history of hospitalization was associated with higher odds of testing positive for MRSA nasal carriage. These results underscored the robustness of the observed association, as they consistently indicated an elevated risk of MRSA nasal carriage across various analytical methods. Additionally, as depicted in Figure 2, the effect size demonstrated directional homogeneity across most stratified analyses, suggesting the general stability of the association between prior hospitalization and MRSA nasal carriage. Furthermore, an E-value was calculated to evaluate the sensitivity to unmeasured confounding, revealing that the primary findings remained robust unless affected by an unmeasured confounder with an OR >3.48.

## 4. Discussion

This cross-sectional study identified an association between prior hospitalization within the past 12 months and an increased risk of MRSA nasal carriage in community-dwelling U.S. adults. The primary finding indicated that, after adjusting for multiple covariates, the OR was 2.03 (95% CI: 1.35–3.05, *P* < .001). PS analyses corroborated these results, yielding an OR of 2.06 (95% CI: 1.37–3.10, *P *< .001). To the best of our knowledge, this study provided novel population-level evidence demonstrating prior hospitalization was independently associated with MRSA nasal carriage in community-dwelling adults, specifically addressing a gap in existing literature predominantly confined to inpatient settings or occupation-specific populations. Furthermore, this investigation stands as the first to have applied PS analysis in evaluating this prior hospitalization–MRSA carriage association among nationally distributed non-institutionalized adults. Nevertheless, further research is necessary to validate these findings.

Previous studies of MRSA nasal carriage have focused largely on hospital-based populations. For example, a prospective multicenter study across 11 German rehabilitation centers involving 5896 non-acute patients demonstrated that hospitalization within the preceding 6 months was independently associated with an elevated risk of MRSA nasal carriage (OR: 2.46, 95% CI: 1.16–5.19, *P* < .019).^[7]^ Similarly, a prospective multicenter study of 2901 surgical inpatients admitted to 13 European wards demonstrated that recent hospitalization within 1 year independently increased the likelihood of MRSA carriage (OR: 2.2, 95% CI: 1.5–3.3, *P *< .001).^[33]^ Additionally, a U.S. hospital-based case–control analysis (n = 156; 78 cases vs 78 matched controls) found that prior hospitalization within 8 years before admission elevated colonization risk (OR: 2.50, 95% CI: 1.15–5.44, *P* = .02).^[8]^ Notably, recent investigations in non-hospitalized populations have observed the association between prior hospitalization and MRSA carriage. A cross-sectional study of Ethiopian university students reported significantly higher MRSA colonization rates among individuals with a hospitalization history (adjusted *P* < .001),^[5]^ while analyses of military personnel revealed prior hospitalization as a strong association of colonization (OR: 2.0, 95% CI: 1.24–3.29, *P* = .005).^[9]^ However, such investigations often involved institutionalized populations (e.g., student or occupation-specific groups). By contrast, our analysis leveraging the NHANES data focused on community-dwelling adults extends these findings beyond institutionalized populations.

Our findings extended the previously documented association between prior hospitalization and MRSA carriage to community-dwelling adults. By utilizing NHANES data that inherently excluded institutionalized individuals, we successfully delineated the associations specifically applicable to community-dwelling adults. After controlling for sociodemographic and clinical confounders, the adjusted odds ratio (OR: 2.03, 95% CI: 1.35–3.05, *P* < .001) was comparable with estimates in institutionalized populations, indicating preserved effect directionality in both community and institutionalized populations. The subtle differences in effect sizes between our community-based estimates and those from previous studies on institutionalized populations could be explained by population-level variations in healthcare contact density, environmental exposures, and/or host susceptibility factors. Moreover, stratified analyses identified a statistically significant interaction by sex (*P* for interaction = .035). Among males, prior hospitalization was associated with a markedly elevated OR of 3.83 (95% CI: 1.79–8.22, *P* < .001), whereas no significant association was observed in females (OR: 1.40, 95% CI: 0.78–2.51, *P* = .266). This cross-sectional study identified a significant association between prior hospitalization and MRSA carriage in community-dwelling populations. The observed sex differences in effect estimates merit further investigation through longitudinal studies.

This association in community-dwelling adults may be linked to hospital-acquired carriage that persists after discharge. MRSA transmission in healthcare facilities may involve by contaminated surfaces (e.g., bedrails) and contact with healthcare workers, potentially leading to persistent colonization during admission.^[34]^ Such colonization may remain unresolved post-discharge, contributing to carriage in non-institutionalized populations.^[4,35]^ This aligns with our findings linking recent hospitalization to MRSA carriage in non-institutionalized adults. Notably, the male-predominant risk elevation may arise from both biological and behavioral determinants. Clinical evidence indicates that males have a higher incidence of MRSA bacteremia compared to females.^[36]^ Specifically, studies report that hospitalized male patients receive more systemic antibiotic courses and undergo invasive procedures more frequently than females, increasing their exposure to nosocomial MRSA transmission.^[37]^ Additionally, emerging evidence has identified that male susceptibility to testosterone-modulated deficits in neutrophil complement receptor 3 expression and priming efficiency may contribute to persistent nasal colonization through impaired pathogen recognition and oxidative burst capacity.^[38,39]^ Collectively, the present study is based on the NHANES database and may provide substantive insights into the association between a history of hospitalization within the past 12 months and the risk of MRSA nasal carriage among community-dwelling U.S. adults.

The present study also has the following limitations. First, the use of a cross-sectional design restricts our ability to establish causality, as it only provides observational information on the association between prior hospitalization and MRSA nasal carriage. To address this limitation, we recommend future longitudinal analyses or experimental designs to more definitively elucidate causal relationships. Second, the cross-sectional nature of the study increases the likelihood of unmeasured confounding. As shown in Table 1, significant differences in sociodemographic characteristics, lifestyle behaviors, and health conditions were observed between hospitalized and non-hospitalized groups. These differences may reflect unmeasured confounders, such as hospitalization duration, which could influence MRSA carriage risk.^[40]^ Although we rigorously adjusted for demographic and clinical covariates, residual confounding may persist. Therefore, we employed E-value sensitivity analysis to assess the potential impact of unmeasured confounders and determined that an unmeasured confounder is unlikely to fully account for the observed association. Third, prior hospitalization data were ascertained via self-reporting, potentially introducing recall bias (e.g., underreporting of brief admissions) or misclassification if participants misunderstood the “overnight” criterion. If these errors occurred non-differentially across exposure groups, the resultant bias would attenuate effect estimates toward the null, suggesting that the actual association is likely stronger than observed. Fourth, missing data for some variables may have introduced bias. However, we applied modern statistical techniques to minimize its impact. Finally, the generalizability of our findings may be limited to community-dwelling U.S. adults. Replication in broader populations would help confirm the observed associations.

## 5. Conclusion

This study analyzed data from the NHANES database focusing on non-institutionalized individuals and identified 9512 participants. It found that prior hospitalization within the past 12 months was associated with an increased risk of MRSA nasal carriage in community-dwelling U.S. adults. Future research could further elucidate potential biological or behavioral mechanisms linking prior hospitalization to MRSA nasal carriage, and multi-regional prospective studies may help assess the consistency of this association.

## Acknowledgments

NHANES data collection was supported by the Centers for Disease Control and Prevention. We wish to convey our sincere appreciation for the steadfast efforts of the NHANES team in the collection, organization, and maintenance of this invaluable database.

## Author contributions

**Conceptualization:** Fengling Zhou, Li Tan.

**Data curation:** Lei Yu, Shaomin Chen.

**Methodology:** Fengling Zhou, Shaomin Chen.

**Software:** Fengling Zhou, Lei Yu.

**Supervision:** Yumei Dong, Li Tan.

**Writing – original draft:** Fengling Zhou, Shaomin Chen.

**Writing – review & editing:** Yumei Dong, Li Tan.
